# Integrated Single‐Cell RNA‐Seq Reveals Immunosuppressive Mechanisms of Treg Cell Differentiation and Tumor Microenvironment Interactions in Colorectal Cancer

**DOI:** 10.1002/cam4.71202

**Published:** 2025-09-02

**Authors:** Dongwei Li, Jingya Liu, Lixian Yu, Xina Luo, Xiaomei Wu, Ronghui Chen

**Affiliations:** ^1^ Dongguan Tungwah Hospital Dongguan Guangdong Province China; ^2^ BioMega Medical Technology Co. Ltd. Zhuhai Guangdong Province China; ^3^ The First Affiliated Hospital of Guangdong Pharmaceutical University Guangzhou Guangdong Province China; ^4^ Department of Anesthesiology Puning People's Hospital Jieyang Guangdong Province China; ^5^ Guangzhou Huaxia Vocational College Guangzhou Guangdong Province China

**Keywords:** colorectal cancer, immune suppression, scRNA‐seq, Treg, tumor microenvironment

## Abstract

**Background:**

Colorectal cancer (CRC) remains a major challenge in oncology, necessitating the identification of new therapeutic targets. This study aimed to explore the CRC microenvironment and uncover potential targets using single‐cell RNA sequencing (scRNA‐seq).

**Methods:**

Single‐cell RNA sequencing data from GEO (GSE164522, GSE132465, and GSE144735) were integrated and stratified based on CRC clinical stages and tumor grades. Cell clustering analysis identified distinct cellular subsets, while survival and functional enrichment analyses evaluated the prognostic significance and biological activity of differentially expressed genes. Additionally, transcription factor activity and pseudotime trajectory analyses elucidated cellular dynamics during CRC progression. Cell–cell communication analysis identified key ligand‐receptor interactions involved in immune regulation. Finally, multiplex immunofluorescence was used to validate the expression of key markers and cells in CRC tissues from patients.

**Results:**

In the low differentiated colorectal adenocarcinoma (LDCA) grade of CRC, the proportion of T cells significantly increased, accompanied by the enrichment of Treg cells and the upregulation of genes associated with immunosuppression, proliferation, and differentiation. Notably, TGFβ1^+^ Tregs significantly expanded in LDCA, accumulating in late tumor grades with enhanced immunosuppressive capacity and self‐proliferation. Pseudotime analysis confirmed their dominance in late CRC, reinforcing immunosuppression through signal amplification and sustained expansion. Concurrently, CD8^+^ T cells in the LDCA grade exhibited a progressive loss of cytotoxic function, transitioning into an exhausted state while concurrently activating apoptotic pathways, leading to a profound impairment of antitumor immunity. Cell–cell communication analysis further demonstrated that TGFβ1^+^ Treg exhibits the strongest interactions with CD8^+^ T cells, with KLRB1‐CLEC2D, LGALS9‐HAVCR2, and TNFSF10‐TNFRSF10B emerging as pivotal ligand‐receptor pairs displaying significantly enhanced signaling in the LDCA grade.

**Conclusion:**

Collectively, TGFβ1^+^ Treg may mediate CD8^+^ T cell dysfunction through these ligand‐receptor interactions, accelerating T cell exhaustion and apoptosis, thereby fostering a profoundly immunosuppressive tumor microenvironment that ultimately drives CRC immune evasion and malignant progression.

## 1 Introduction

Colorectal cancer (CRC), characterized by its high incidence and mortality rates, remains a formidable challenge in clinical treatment, particularly for patients with advanced or drug‐resistant disease [1, 2]. Despite the availability of various therapeutic modalities, including surgery, radiotherapy, chemotherapy, targeted therapy, and immunotherapy, effective treatment remains elusive, and overall prognosis remains poor. In recent years, single‐cell RNA sequencing (scRNA‐seq) has emerged as a pivotal technology in cancer research, enabling the elucidation of cellular heterogeneity within the tumor microenvironment and the dissection of key mechanisms underlying immunotherapeutic responses [3, 4, 5]. This advanced technique not only deepens our understanding of immune evasion in CRC but also provides novel insights for optimizing personalized therapeutic strategies [6, 7, 8].

When discussing immunotherapeutic strategies for CRC, it is essential to highlight the role of the tumor microenvironment (TME) [9]. Within the TME, as CRC cells undergo dedifferentiation, they exhibit increasing abnormalities, lose their normal structural characteristics, and acquire a higher degree of malignancy. Simultaneously, immune cells within the TME also interact with one another, and these interactions can actively suppress antitumor immune responses, thereby facilitating immune evasion by tumor cells [10, 11]. Notably, regulatory T cells (Tregs) represent a distinct subset of immunosuppressive cells that exhibit remarkable phenotypic and functional heterogeneity within the TME, particularly in CRC. Tregs primarily contribute to tumor progression, immune evasion, and metastasis by secreting inhibitory cytokines and directly suppressing effector T‐cell activity [12, 13]. The accumulation of Tregs in the TME is strongly associated with tumor progression, immune evasion, and metastatic dissemination [14]. Given their pivotal role in immune suppression, Tregs have been increasingly recognized as a crucial target for CRC immunotherapy. Current therapeutic strategies aimed at counteracting Treg‐mediated immunosuppression include Treg depletion, functional reprogramming, or inhibition, all of which seek to restore antitumor immune responses and enhance therapeutic efficacy [15, 16, 17].

CD8^+^ T cells are critical effector cells in tumor immunity, responsible for recognizing and eliminating tumor cells. However, Tregs suppress CD8^+^ T cell activity through multiple mechanisms, leading to immune evasion [18]. For instance, Tregs highly express CTLA‐4, which binds to CD80/CD86 on antigen‐presenting cells, thereby blocking co‐stimulatory signaling and impairing CD8^+^ T cell activation [19, 20]. In CRC, Tregs further inhibit CD8^+^ T cell function through specific receptor‐ligand interactions, particularly within the TME. These interactions not only diminish CD8^+^ T cell proliferation and cytotoxicity but also promote their functional exhaustion. For example, studies have demonstrated that Tregs interact with CD8^+^ T cells via the TIGIT/CD155 axis, suppressing IFN‐γ and TNF‐α secretion and weakening their antitumor capacity. Additionally, Tregs express the CCR4 receptor, which binds to CCL22 secreted by tumor cells, facilitating Treg recruitment into the TME and enhancing localized immunosuppressive effects [21]. As CRC progresses, Tregs undergo specific differentiation and form distinct subsets to adapt to the evolving immune landscape. For example, thymus‐derived Tregs (tTregs) primarily develop in the thymus, maintaining self‐tolerance and preventing autoimmune reactions [22]. Additionally, NCF1^+^ Tregs manage oxidative stress responses, protecting tumor cells from oxidative damage [23]. Proliferative Tregs have a high proliferative capacity, expanding rapidly in early tumors to suppress initial anti‐tumor immune responses. HSP^+^ Tregs are linked to stress responses, secreting stress proteins to help tumor cells resist stress‐induced cell death, thus enhancing tumor cell survival, especially in poorly differentiated tumors [24, 25]. Notably，TGFβ1^+^ Tregs play a pivotal role in shaping the immunosuppressive tumor microenvironment in CRC. By secreting TGF‐β1, these Tregs not only potentiate their own suppressive capacity but also effectively attenuate the activity of effector T cells, thereby dampening antitumor immune responses [26, 27]. Recent studies have shown that the high infiltration of Tregs with elevated TGFβ1 expression in CRC tissues is not only closely associated with poor patient prognosis but also linked to increased resistance to PD‐1/PD‐L1 immune checkpoint inhibitors [28, 29]. Collectively, these findings emphasize the central immunoregulatory function of TGFβ1^+^ Tregs in CRC progression, highlighting them as potential therapeutic targets for modulating the tumor microenvironment and restoring antitumor immunity.

Therefore, Tregs, particularly their various subtypes, play a crucial immunoregulatory role in the progression of colorectal cancer. To elucidate the immune mechanisms underlying CRC progression, we integrated scRNA‐seq data from three GEO databases to systematically investigate intercellular signaling and interactions within the tumor and its microenvironment. Our study aims to uncover the specific functions and dynamic changes of Tregs in CRC immune evasion, analyze their interaction networks with effector T cells and other immune cells, and identify key immunosuppressive pathways. Through these analyses, we seek to provide a deeper theoretical foundation for remodeling the CRC immune microenvironment and explore potential immunotherapeutic targets to improve treatment strategies and patient prognosis.

## 2 Methods

### 2.1 Data Selection

Three publicly available scRNA‐seq datasets (GSE164522, GSE132465, and GSE144735) were selected from the Gene Expression Omnibus (GEO) database. These datasets were chosen based on the following criteria: (1) a large sample size, which ensures sufficient statistical power for analysis; (2) high data quality, with stringent filtering criteria such as nUMI, nGene, and mitochondrial gene percentage; and (3) strong clinical relevance, as they include CRC patients with detailed stage and grade classifications, covering both primary and metastatic tumors. The integration of these datasets enables a comprehensive analysis of immune cell heterogeneity in CRC progression.

### 2.2 scRNA Data Preprocessing, Dimensionality Reduction, and Clustering

Most analyses were conducted utilizing the python package “scanpy” (version 1.10.2) [30]. The initial preprocessing steps were as follows: (a) loading datasets GSE164522, GSE132465, and GSE144735, with all patient information after dataset integration as shown in Table S1; (b) filtering out cells with counts above 700, gene numbers greater than 500, and mitochondrial gene proportions under 20% [31]; and (c) removing doublets with Scrublet [32]. After filtering, gene expression data were normalized, scaled, and log transformed. The “highly_variable_genes” function was used to identify the top 2000 highly variable genes from the dataset. Preprocessing concluded by centering and scaling the expression data.

Following preprocessing, dimensionality reduction was executed using Principal Component Analysis (PCA) through the “pca” function. The integration result is shown in Figure S1A. Data integration was then performed in the reduced PCA space using the “harmony” function. Neighbor analysis was conducted by selecting the top 50 PCA components with the “neighbors” function, followed by clustering visualization in the Uniform Manifold Approximation and Projection (UMAP) space utilizing “umap” and “leiden” functions. After identifying cell clusters, these clusters were grouped based on gene expression and distribution similarity. Specific marker genes were visualized using the “dotplot” function. The final scRNA dataset was prepared for further downstream analyses, including trajectory analysis and cell–cell communication inference.

### 2.3 Survival Analysis

Survival analyses were performed on TCGA colorectal cancer data using the Survival package (version 3.7). Samples were classified into the high expression group if their gene expression levels were in the top 30%, and into the low expression group if their gene expression levels were in the bottom 30% [33]. The survfit function was used to perform Kaplan–Meier survival analysis, with a *p*‐value of < 0.05 considered significant.

### 2.4 Gene Ontology (GO) Enrichment Analysis

Differentially expressed genes (DEGs) were identified using the “FindMarkers” function in the Seurat R package, applying the Wilcoxon Rank Sum test with Bonferroni correction. Genes with an adjusted *p*‐value < 0.05 and absolute log2FC > 0.25 were selected for further analysis. Samples were grouped based on tumor differentiation, with LDCA (low differentiation grade) compared against the combined HDCA (high differentiation grade) and MDCA (medium differentiation grade). DEGs were identified as genes significantly upregulated in LDCA relative to the combined groups.

Gene Ontology (GO) enrichment analysis was performed using the “enrichGO” function in clusterProfiler, and results with FDR ≤ 0.05 were visualized using ggplot2.

### 2.5 Differential Gene Analysis

For differential gene expression analysis, the criteria were avg_log2FC > 0.25 and *p*‐val < 0.05. GO database gene set enrichment analysis was performed using ClusterProfiler. Survival analysis of the identified genes was conducted using TCGA COAD data. SCENIC analysis using the pySCENIC package was performed on Treg cells.

### 2.6 Transcriptional Regulatory Network Inference

Inference of single‐cell transcription factor (TF) regulatory networks was performed using pySCENIC (version 0.12.1) [34]. A TF database, derived from the hg38 genome, was chosen and downloaded, with the search space encompassing 500 bp upstream to 100 bp downstream of the transcription start site (TSS). TF enrichment was carried out using AUCell, and the spatial distribution of enriched TFs was depicted in a UMAP‐based plot. The database employed for this analysis can be accessed at https://resources.aertslab.org/cistarget.

### 2.7 Cell–Cell Communication Network Inference

The interactions between epithelial cells and other cell types were examined utilizing the R package “CellChat” (version 2.1.2). Employing the ligand‐receptor pair database within the “Secreted Signaling” category, the subsequent analyses were performed as follows: (1) evaluating communication probability and deducing communication networks with the “computeCommunProb” and “filterCommunication” functions; (2) assessing pathway‐level communication probability using the “computeCommunProbPathway” function; and (3) constructing aggregated cell–cell communication networks via the “aggregateNet” function.

### 2.8 Pseudotime Analysis

Pseudotime analysis was performed using Monocle2 (version 2.9.0) [35]. To normalize the data, the “estimateSizeFactors” and “estimateDispersions” functions were utilized, accounting for differences in sequencing depth and gene expression dispersion. Genes were filtered by retaining those with a minimum expression threshold of 0.1 using the “detectGenes” function. A dispersion table was generated using the “dispersionTable” function, selecting genes with a mean expression above 0.1. The “setOrderingFilter” function was applied to filter genes for trajectory inference, followed by dimensionality reduction through the “reduceDimension” function. The “orderCells” function was used to infer cell trajectories, uncovering dynamic changes in cell development, differentiation, or disease progression.

### 2.9 Single‐Sample Gene Set Enrichment Analysis (ssGSEA)

Gene sets obtained from the MSigDB database covered immune suppression, cell migration, antigen presentation suppression, cell differentiation, and cell survival and metabolism. ssGSEA was applied to assess the activity of different Treg cell subsets across these functional pathways.

### 2.10 Patient Sample Collection

Paraffin‐embedded samples from CRC patients were obtained from our hospital, with different grades of the patients identified by hospital pathologists. The study was approved by the Ethics Committee under approval number 2017DHLL019. Our research adhered to ethical guidelines, ensuring that all patients provided informed consent.

### 2.11 Multiplex Immunohistochemistry

Paraffin‐embedded patient tissue sections (5 μm) were first deparaffinized using xylene, followed by rehydration with a graded ethanol series. The sections were then placed in pH 9.0 EDTA antigen retrieval buffer and subjected to high‐pressure antigen retrieval for 2 min, followed by natural cooling for 30 min. The sections were washed with PBS three times, each for 5 min. Endogenous peroxidase activity was blocked by incubating the sections with 3% H₂O₂ solution in the dark for 15 min. After PBS washing, the sections were incubated with blocking solution containing 3% BSA and 0.1% Triton X‐100 at 37°C for 30 min. Subsequently, a tyramide signal amplification (TSA) staining procedure was performed. First, the sections were incubated overnight at 4°C with rabbit anti‐FOXP3 primary antibody (1:100). After PBS washing, HRP‐conjugated goat anti‐rabbit secondary antibody was applied and incubated at 37°C for 30 min. Tyramide‐Cy3 working solution was then applied and incubated for 5 min in the dark. After PBS washing, the antibody was stripped using glycine‐HCl buffer (pH 2.0) for 10 min. Next, CD3 staining was performed by incubating the sections with mouse anti‐CD3 primary antibody (1:150) at 37°C for 1 h. After PBS washing, HRP‐conjugated goat anti‐mouse secondary antibody was applied, followed by TSA‐AF488 for signal amplification. Lastly, TGFβ1 staining was carried out by incubating the sections with rabbit anti‐TGFβ1 primary antibody (1:200) at 37°C for 2 h. After PBS washing, HRP‐conjugated secondary antibody was applied, followed by TSA‐Cy5 for signal amplification. After the final staining, the nuclei were counterstained with DAPI (1 μg/mL) for 5 min. Following PBS washing, the sections were mounted with anti‐fade mounting medium (ProLong Diamond), and images were captured and stored.

### 2.12 Statistical Analyses

All differential gene expression analyses were conducted using R (version 4.3.1), and statistical significance was evaluated using two‐tailed rank‐sum test *p*‐values. In our study, *p*‐values < 0.05 were considered statistically significant.

## 3 Results

### 3.1 High‐Resolution Landscape of All Cell Types in CRC

**FIGURE 1:**
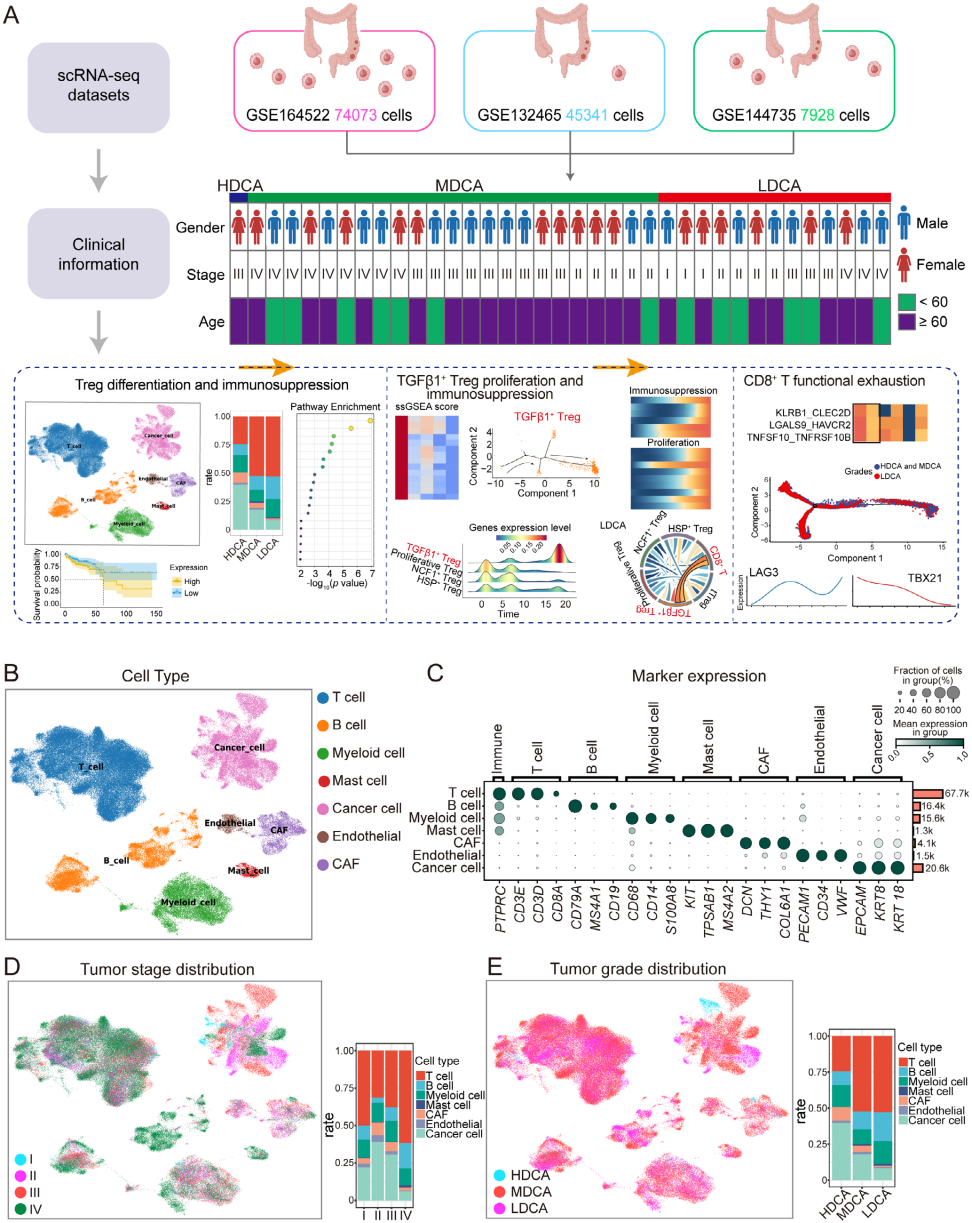
High‐resolution landscape of all cell types in colorectal cancer. (A) Flowchart of the experimental design, showing the overall process of data collection and analysis. (B) UMAP plot of all colorectal cancer single cells, colored by cell type. (C) Bubble plot showing the expression of cell type‐specific marker genes in different cells. The larger the bubble, the higher the cell proportion, and the deeper the green, the higher the gene expression level. (D) UMAP plot of colorectal cancer single cells colored by tumor progression stage, showing the proportion of each cell type. (E) UMAP plot of colorectal cancer single cells colored by tumor differentiation grade, showing the proportion of each cell type.

We integrated a total of 127,342 single cells from colorectal cancer (CRC) samples collected from 43 patients across three publicly available GEO datasets, namely GSE132465, GSE144735, and GSE164522 (Figure S1A). After stringent quality control and filtering, we included only those patients with complete tumor stage and pathological grade information for downstream analysis (Figure 1A). Clustering of these cells revealed the presence of seven major cell types, including T cells, B cells, myeloid cells, mast cells, cancer cells, endothelial cells, and cancer‐associated fibroblasts (CAFs) (Figure 1B). The expression patterns of marker genes across different cell types are depicted in the dot plot, where bubble size represents the relative proportion of each cell type within the sample, while color intensity reflects gene expression levels (Figure 1C). To explore the potential associations between different cell types and TNM staging or tumor differentiation status, we first performed clustering analysis based on tumor differentiation grade and compared the proportions of various cell types across different TNM stages. The results showed no significant correlation between the proportion of cell types and CRC staging (Figure 1D). Based on the pathological grading information provided in the clinical annotations of the original GEO datasets, we categorized the samples into three groups defined as highly differentiated (HDCA), moderately differentiated (MDCA) and low differentiated (LDCA), which formed the basis for subsequent single‐cell analyses. Across these differentiation grades, tumor cell malignancy exhibited a gradient increase from low to high, where HDCA tumors consisted of highly differentiated cancer cells with lower malignancy, MDCA tumors exhibited intermediate differentiation and moderate malignant potential, while LDCA tumors were characterized by low differentiation, high proliferative activity, and enhanced invasiveness [36].

Interestingly, as CRC differentiation decreased (progressing from HDCA to LDCA), the proportion of T cells significantly increased, whereas the proportion of malignant tumor cells relatively declined, forming a distinct contrast (Figure 1E). This finding suggests that, compared to TNM staging, tumor differentiation status may exert a greater influence on the dynamic regulation of the immune microenvironment. As tumor differentiation decreases, the regulatory mechanisms governing immune cell composition appear to become increasingly intricate, reflecting a progressive reprogramming of the tumor microenvironment during CRC progression.

### 3.2 Immune Suppression and Proliferative Dynamics of Treg Cells in LDCA Grade of CRC

**FIGURE 2:**
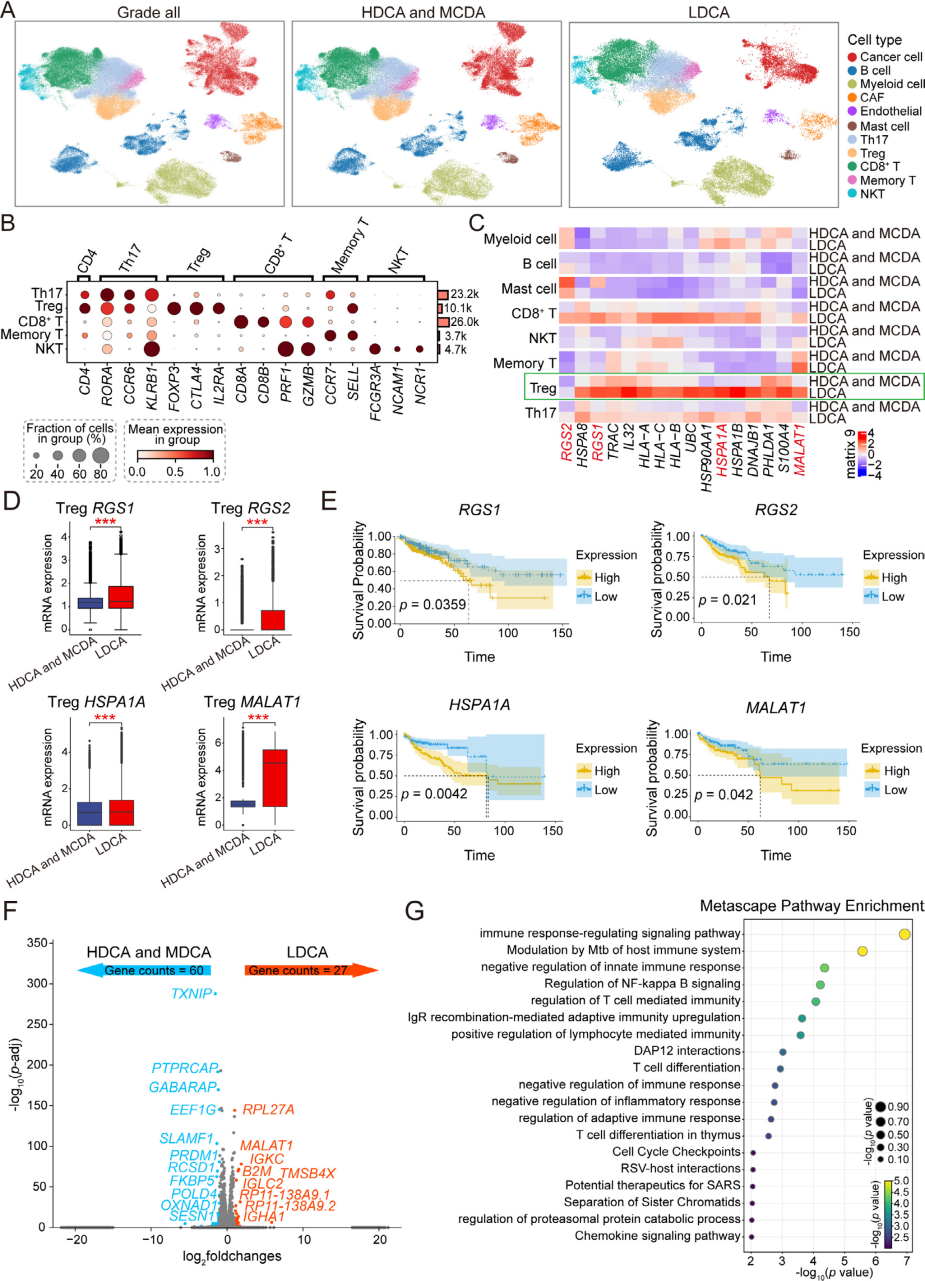
Functional analysis of treg cells across different grades. (A) UMAP plot of reclustered cell distribution after merging HDCA and MDCA samples. (B) Expression of cell type‐specific marker genes, where dot size represents the proportion of the cell type among all cells, and deeper red indicates higher average gene expression levels. (C) Heatmap of gene expression levels in different cells comparing HDCA and MDCA grade to LDCA grade, where redder colors indicate higher average gene expression (log FC ≥ 0.25, adjusted *p*‐value ≤ 0.05). (D) Box plots comparing the expression levels of *RGS1*, *RGS2*, *HSPA1A*, and *MALAT1* in Treg cells at different differentiation grades in CRC, where *** indicates *p* < 0.001. (E) Survival analysis of *RGS1*, *RGS2*, *HSPA1A*, and *MALAT1* in the TCGA COAD dataset. (F) Volcano plot of differentially expressed genes between the two grades. (G) Pathway enrichment analysis of highly expressed genes in LDCA compared to other grades.

To scientifically and accurately reflect the data, and considering the limited sample size of HDCA patients, we combined the data of HDCA and MDCA patients for comparison with LDCA patients. Ensuring scientific rigor, we re‐clustered all colorectal cancer cells based on these two grades and identified distinct cell types by analyzing the high expression levels of cell type‐specific marker genes (Figure 2A,B). During the analysis of these cells, we observed that Treg cells exhibited significantly elevated expression of multiple genes closely associated with immune suppression in CRC during the LDCA grade. Tregs are a subset of T cells with immunosuppressive functions, playing a crucial role in immune regulation during tumor immune responses. For instance, RGS1 has been identified as a novel marker and promoter of CD8^+^ T cell exhaustion, while RGS2 is closely linked to the distribution and function of tumor‐infiltrating immune cells within the CRC tumor microenvironment [36, 37, 38]. Additionally, *HSPA1A*, a heat shock protein, plays a crucial role in promoting tumor cell survival and chemoresistance, enhancing cancer cell adaptability and stress resistance. Its low expression correlates with improved prognosis in CRC patients [39, 40]. Similarly, *MALAT1*, a long non‐coding RNA, not only regulates gene expression and cellular signaling networks but also enhances tumor cell invasivenes [41, 42]. Studies indicate that *MALAT1* is highly expressed in CRC and is closely associated with tumor staging and patient prognosis. RNA interference‐mediated *MALAT1* knockdown effectively suppresses CRC growth and progression [43] (Figure 2C). As expected, we found that these genes were significantly upregulated in the LDCA grade compared to HDCA and MDCA grades, and their high expression in Tregs was associated with poor survival in colorectal cancer patients (Figure 2D,E). Moreover, we found that in CRC patient samples, the number of Treg cells in the LDCA grade was higher than in other grades (Figure S2).

Moreover, a comprehensive comparison of Treg cells in the LDCA, HDCA, and MDCA grades was conducted, focusing onpatterns (Figure 2F). Pathway enrichment analysis was further performed on LDCA‐specific highly expressed genes. The analysis revealed significant functional changes in Treg cells during the LDCA grade, with their regulatory networks not only shifting toward immune suppression but also enhancing self‐proliferation and differentiation (Figure 2G). At the immune regulation level, LDCA‐grade Treg cells were highly enriched in negative immune regulatory pathways, including negative regulation of innate immune response, regulation of T cell mediated immunity, and negative regulation of immune response. Meanwhile, Treg cells exhibited increased activity in pathways related to T cell proliferation and differentiation, such as T cell differentiation and T cell differentiation in thymus. This trend suggests that Treg cells in the LDCA grade not only reinforce immune suppression but may also further consolidate their negative regulation of the immune system by maintaining their differentiation and stability.

### 3.3 TGFβ1 ^+^ Treg in LDCA‐Grade CRC Exhibit Robust Immunosuppressive Activity

**FIGURE 3:**
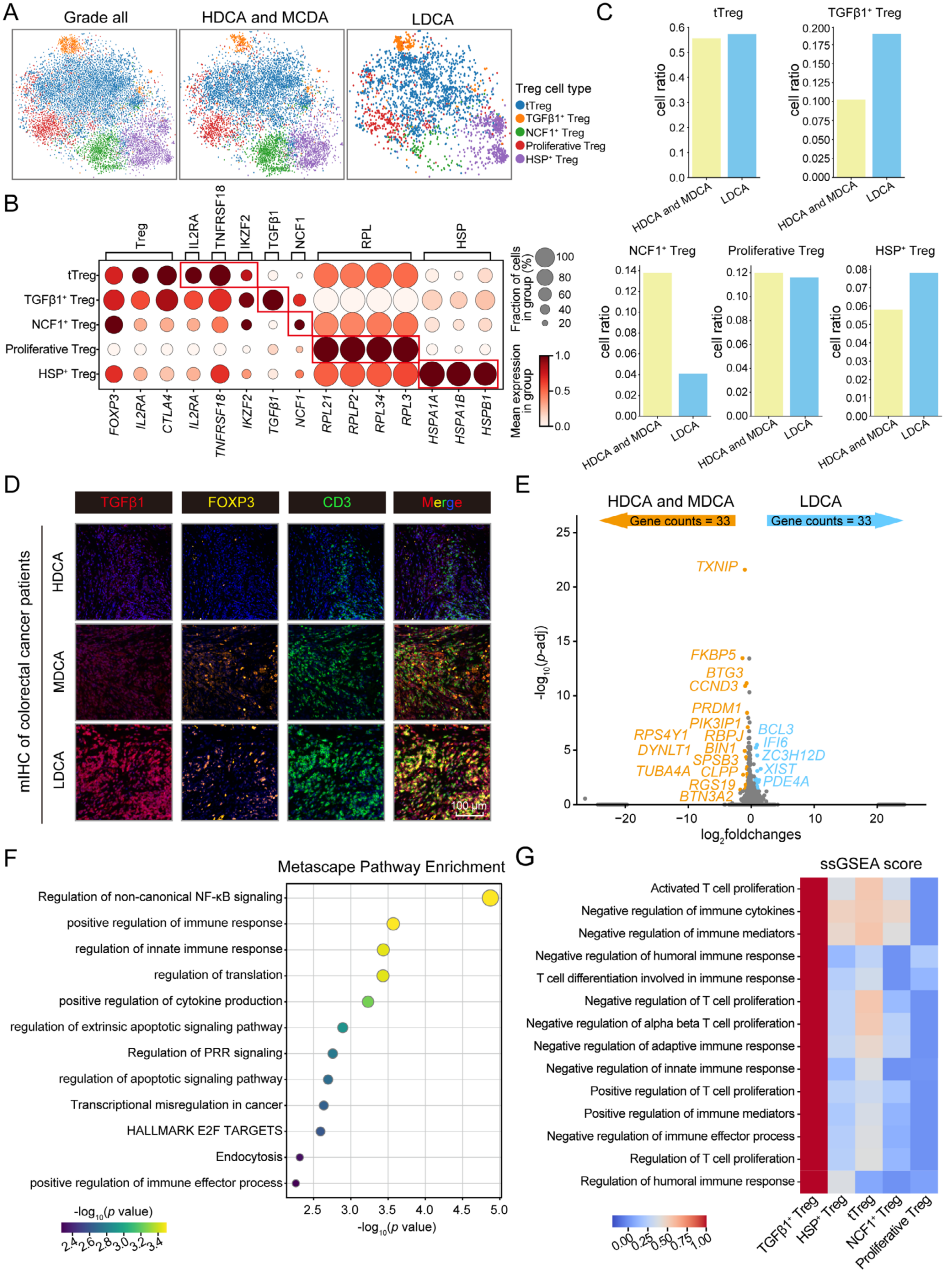
Comprehensive analysis of treg subtypes in relation to colorectal cancer differentiation grades. (A) UMAP plot of Treg subtypes displayed by CRC grades, with five Treg subclasses represented by dots in distinct colors. (B) Expression levels of marker genes in Treg subtypes, where darker colors indicate higher average gene expression, and larger dots represent a higher proportion of corresponding cells. (C) Bar chart showing the proportional changes of Treg subtypes across different CRC grades. (D) Representative immunofluorescence staining images of CRC patient tissue sections for each grade, where TGFβ1 (red), FOXP3 (yellow), CD3 (green) and DAPI (blue) are shown. Scale bar = 100 μm. (E) Volcano plot of differentially expressed genes in TGFβ1^+^ Treg between LDCA and HDCA/MDCA grades. (F) Pathway enrichment analysis of highly expressed genes in TGFβ1^+^ Treg in LDCA grade relative to HDCA and MDCA grades, with darker colors indicating higher significance. (G) SsGSEA pathway scoring for Treg subtypes, where redder colors indicate stronger pathway activity.

The increase in Treg cells and the high expression of genes associated with poor prognosis in the LDCA grade prompted us to investigate whether specific Treg subpopulations play a key role in this process. Initially, we performed batch correction on the single‐cell data and identified several Treg subpopulations based on the high expression of their specific marker genes. These subpopulations include tTreg, TGFβ1^+^ Treg, NCF1^+^ Treg, Proliferative Treg, and HSP^+^ Treg (Figure S1B; Figure 3A,B). During our exploration, we identified a distinct subpopulation, TGFβ1^+^ Treg, which exhibited a notable increase in the LDCA grade (Figure 3C). This change was further validated in CRC patient samples, where the number of TGFβ1^+^ Treg cells increased in the LDCA grade (Figure 3D). Consequently, we conducted differential gene expression analysis of this subpopulation across different grades and performed pathway enrichment analysis using its highly expressed genes in the LDCA grade (Figure 3E,F). The enrichment results indicated that TGFβ1^+^ Treg actively engages with immune response pathways, such as the regulation of non‐canonical NF‐κB signaling, which may influence its development, function, and stability, thereby participating in immune suppression. Additionally, it actively responds to translational regulation pathways, suggesting its involvement in proliferation and differentiation.

Furthermore, we performed ssGSEA on these five Treg subtypes. The resulting scores reflect the activity or expression patterns of specific gene sets within individual cells, aiding our understanding of the heterogeneity and functional differences among the five Treg subpopulations (Figure 3G). Notably, we discovered that, compared to other subtypes, TGFβ1^+^ Treg demonstrates strong activity in the positive regulation of proliferation and negative regulation of immune responses, including activated T cell proliferation, regulation of T cell proliferation, negative regulation of humoral immune response, and negative regulation of adaptive immune response. These findings underscore the significant increase of TGFβ1^+^ Treg in the LDCA grade and their critical role in immunosuppression and proliferative differentiation, highlighting the crucial role of TGFβ1^+^ Treg in the regulatory network of CRC progression.

### 3.4 TGFβ1 ^+^ Treg Facilitate Immunosuppression and Expansion During CRC Progression

**FIGURE 4:**
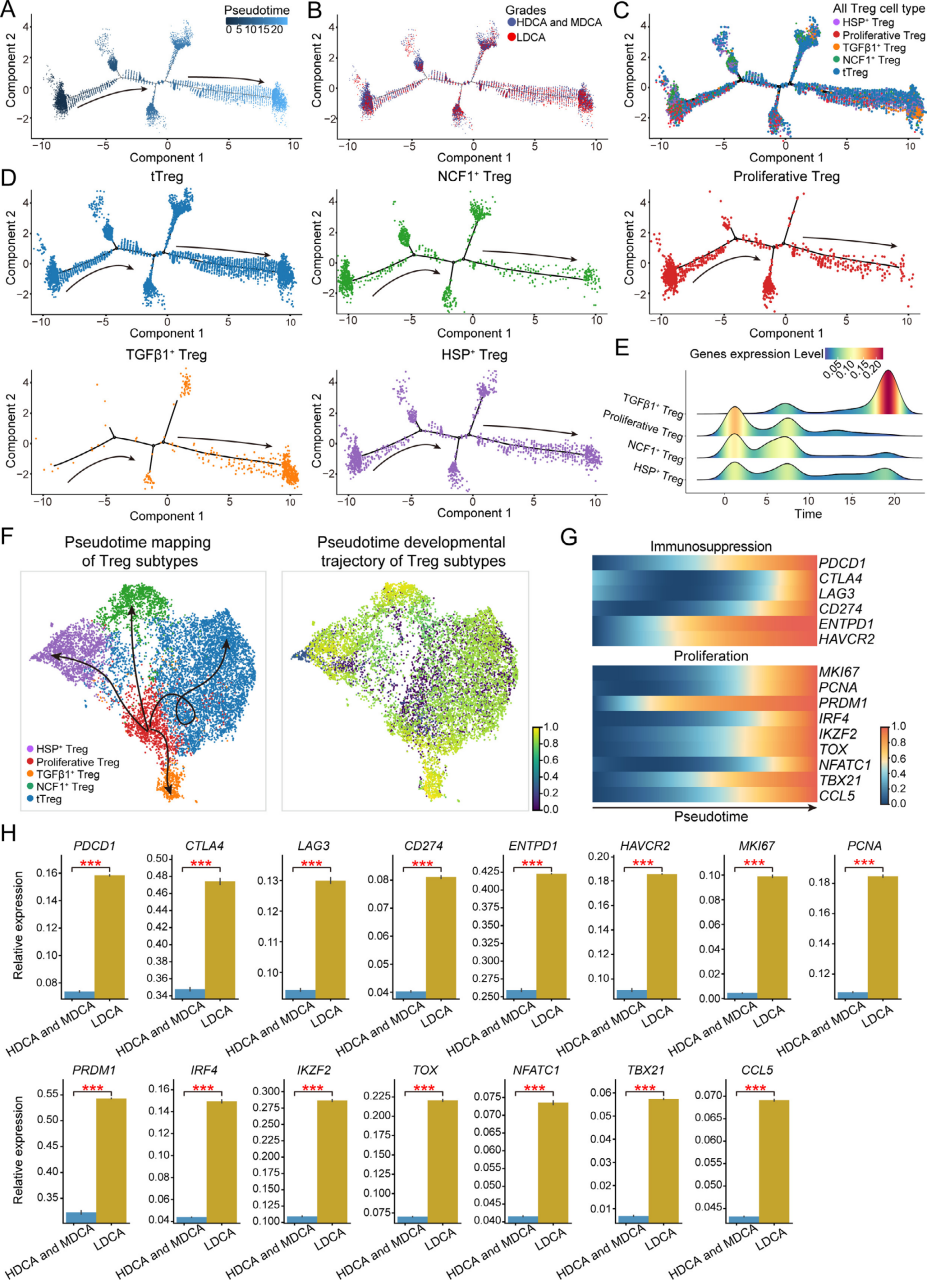
Pseudotime developmental trajectories and gene expression dynamics of Treg subtypes in CRC. (A) Schematic representation of all pseudotime developmental trajectories, with colors transitioning from dark to light indicating the progression of time. (B) Pseudotime developmental trajectories of Treg cells across different CRC grades. (C) Schematic representation of pseudotime developmental trajectories for five Treg subtypes, where each dot represents a different subtype. (D) Individual cell developmental trajectories for each Treg subtype. (E) Gene expression levels across Treg subtypes as a function of cell development, with redder colors indicating higher gene expression levels. (F) Pseudotime developmental trajectories of Treg subtypes along the tumor differentiation gradient from HDCA to LDCA (left), with corresponding developmental time representation (right), where colors range from deep blue to yellow, representing early to late developmental grades. (G) Gene expression dynamics of TGFβ1^+^ Treg along pseudotime, highlighting genes associated with immunosuppression and proliferation, with redder colors indicating higher expression levels. (H) Bar plot showing the proportion of gene expression in TGFβ1^+^ Treg across HDCA and MDCA compared to the LDCA group, where *** indicates *p* < 0.001.

Pseudotime analysis infers the developmental order of cells by calculating transcriptional similarities between them, thereby constructing a pseudotime axis to delineate the dynamic processes of cell differentiation, state transitions, and disease progression. Therefore, we employed pseudotime analysis to investigate the developmental trajectory of Treg cells across different CRC grades. The results revealed that in the LDCA grade, Treg cells were predominantly distributed in the later grades of differentiation, suggesting that they might be in a more mature or functionally enhanced state (Figure 4A,B). To further clarify the roles of Treg subpopulations in this process, we conducted pseudotime analysis on each Treg subset to characterize their developmental trajectories. Distinct differences emerged in the distribution patterns among the subsets, with TGFβ1^+^ Treg being highly enriched at the later grades of differentiation (Figure 4C,D). This subset not only exhibited a strong presence in the late developmental phase but also showed a generally higher expression level of crucial genes compared to other subtypes (Figure 4E). To explore the functional evolution of TGFβ1^+^ Treg during CRC grade transitions, we visualized the pseudotime developmental trajectories of different Treg subsets from the HDCA to LDCA grade (Figure 4F). Heatmap analysis revealed that in the later grades of CRC, the immunosuppressive function of TGFβ1^+^ Treg was progressively enhanced. Several classical immune inhibitory molecules, including *PDCD1*, *CTLA4*, and *LAG3*, exhibited significantly elevated expression in the LDCA grade (Figure 4G). The sustained upregulation of these genes suggests that TGFβ1^+^ Treg may actively suppress CD8^+^ T cell proliferation and activation, thereby attenuating antitumor immune responses and facilitating immune evasion. Moreover, changes in proliferation‐associated genes within TGFβ1^+^ Treg warrant attention. Crucial molecular drivers such as *MKI67*, *PCNA*, and *IRF4* were highly expressed in the later developmental grades (Figure 4G). *MKI67* serves as an indicator of cell cycle progression, *PCNA* is a critical regulator of DNA replication and cell proliferation, and *IRF4* plays a pivotal role in Treg differentiation and immunosuppression [44]. The activation of proliferative signals suggests that TGFβ1^+^ Treg not only increase in number but also expand their functional influence within the tumor microenvironment.

Notably, both immune suppression and proliferation‐associated genes exhibited significantly higher expression in the LDCA grade compared to the HDCA and MDCA grades (Figure 4H). This indicates that during CRC progression, TGFβ1^+^ Treg undergo profound biological transformations, shifting from a component of homeostatic immunity to a highly active immunosuppressive population. As their influence in the immune microenvironment grows, they not only enhance immunosuppressive activity, weakening antitumor immune responses, but also exhibit increased proliferative capacity, allowing for continuous expansion. These changes collectively reinforce their role in shaping an immunosuppressive tumor environment and driving disease progression.

### 3.5 TGFβ1 ^+^ Treg Exhibits the Potential to Drive CD8 ^+^ T Cell Dysfunction Through Critical Ligand‐Receptor Interactions

**FIGURE 5:**
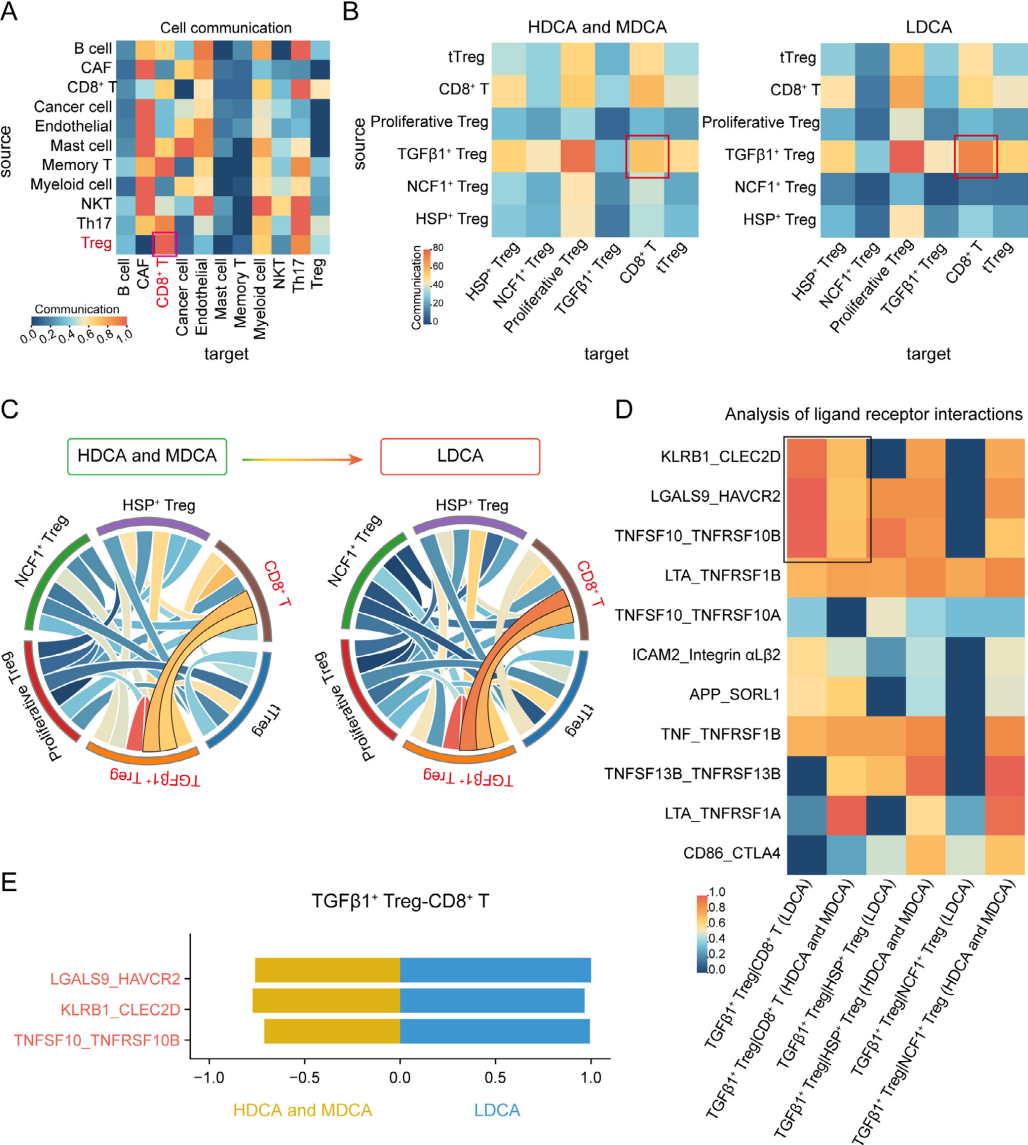
Analysis of cell–cell interactions. (A) Analysis of intercellular interactions among all cell types, with redder colors indicating stronger signaling intensity. (B) Heatmap illustrating the interaction strength between Treg subpopulations and CD8^+^ T cells across different CRC differentiation grades, where redder colors denote higher communication intensity. (C) Circular diagram depicting signaling exchanges between Treg subpopulations and CD8^+^ T cells across various CRC differentiation grades, with redder colors reflecting more pronounced interactions. (D) Heatmap representing the strength of ligand‐receptor interactions, where redder colors indicate greater binding affinity. (E) Comparative analysis of TGFβ1^+^ Treg‐CD8^+^ T cell communication in HDCA/MDCA and LDCA.

To explore the potential mechanisms underlying the immunosuppressive function of TGFβ1^+^ Treg, we conducted a cell–cell communication analysis, which revealed that interactions between Treg and CD8^+^ T cells were the most pronounced (Figure 5A). Comparing the signaling strength between five Treg subgroups and CD8^+^ T cells, an intriguing pattern emerged. In contrast to HDCA and MDCA grades, TGFβ1^+^ Treg not only exhibited the strongest autocrine signaling but also exerted the most substantial regulatory influence on CD8^+^ T cells (Figure 5B,C).

As ligand‐receptor interactions serve as the fundamental mechanism driving cell–cell communication, mediating signal transduction and functional regulation through ligand binding, these molecular interactions play a critical role in maintaining immune homeostasis and orchestrating complex biological processes. Among the numerous ligand‐receptor pairs identified, three stood out as key mediators of TGFβ1^+^ Treg‐driven CD8^+^ T cell dysfunction, including KLRB1‐CLEC2D, LGALS9‐HAVCR2, and TNFSF10‐TNFRSF10B. KLRB1‐CLEC2D (CD161‐CLEC2D) exerts immunosuppressive effects by downregulating metabolic activity and impairing the expression of cytotoxic effector molecules in CD8^+^ T cells. Consequently, the inactivation of KLRB1 or antibody‐mediated blockade of CD161 restores T cell metabolic capacity, thereby enhancing cytotoxicity against tumor cells and augmenting systemic antitumor immune responses [45]. Moreover, LGALS9‐HAVCR2 (Galectin‐9‐TIM‐3) serves as a pivotal regulator of T cell exhaustion, with elevated TIM‐3 expression in various malignancies strongly correlating with poor prognosis and functional exhaustion of CD8^+^ tumor‐infiltrating lymphocytes (TILs). This suggests that within the tumor microenvironment, persistent engagement of this pathway may further accelerate CD8^+^ T cell dysfunction, ultimately leading to the loss of antitumor immunity and facilitating immune evasion [46]. Meanwhile, TNFSF10‐TNFRSF10B (TRAIL‐DR5) interaction activates the extrinsic apoptotic cascade, triggering caspase‐mediated signaling that culminates in tumor cell apoptosis [47]. Studies have demonstrated that a DR5‐specific TRAIL variant, DR5‐B, enhances pro‐apoptotic signaling within tumor cells, thereby effectively suppressing CRC progression. These findings underscore the critical role of DR5‐mediated apoptosis in the immunoregulatory landscape of CRC [48]. Notably, these ligand‐receptor interactions exhibited enhanced signaling intensity in the LDCA grade, highlighting their increasing role in modulating CD8^+^ T cell fate as tumor differentiation declines (Figure 5D). Consistently, the overall activity of these pathways was significantly elevated in the LDCA group (Figure 5E). As CRC progresses toward a poorly differentiated state, it is highly likely that TGFβ1^+^ Treg, through these ligand‐receptor interactions, exerts a profound regulatory influence over CD8^+^ T cells.

By driving exhaustion, impairing cytotoxic function, and promoting apoptosis, these interactions establish a potent immunosuppressive network that fosters immune evasion and accelerates disease progression.

### 3.6 TGFβ1 ^+^ Treg Promote CD8 ^+^ T Cell Exhaustion and Cytotoxic Decline in LDCA Grade CRC

**FIGURE 6:**
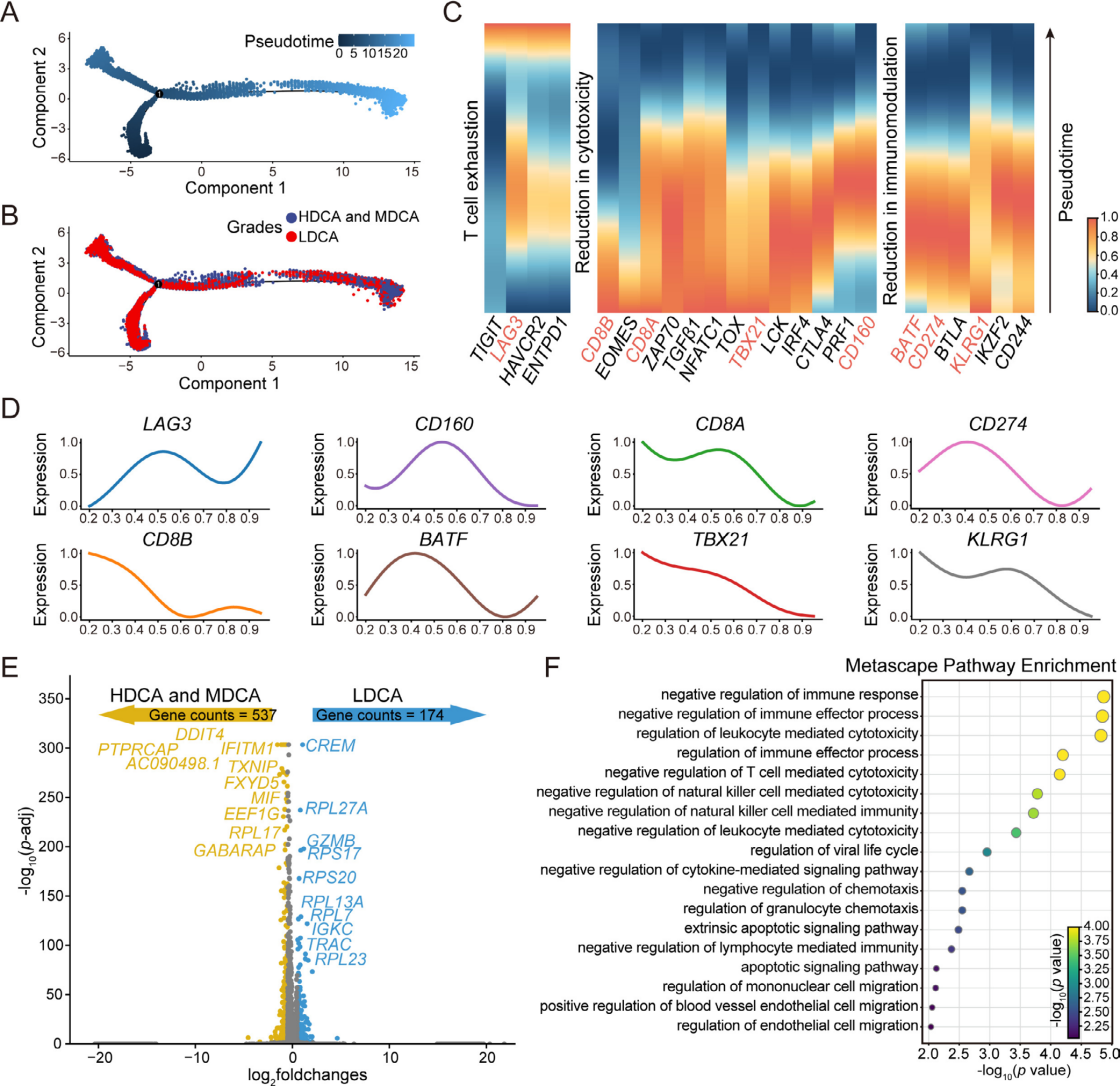
Pseudotime analysis reveals CD8^+^ T cell functional transitions across CRC grades. (A) Schematic representation of all pseudotime developmental trajectories, with colors transitioning from dark to light indicating the progression of time. (B) Pseudotime developmental trajectories of CD8^+^ T cells across different CRC grades. (C) Gene expression dynamics of CD8^+^ T cells during development. (D) Expression levels of selected genes from C during the developmental process. (E) Volcano plot of differentially expressed genes in CD8^+^ T cells between LDCA and HDCA/MDCA grades. (F) Pathway enrichment analysis of highly expressed genes in CD8^+^ T cells in LDCA grade relative to HDCA and MDCA grades, with more yellow colors indicating higher significance.

How does the interaction between TGFβ1^+^ Treg and CD8^+^ T cells influence the functional state of CD8^+^ T cells? It is well established that CD8^+^ T cells play a pivotal role in tumor immune surveillance, primarily through direct cytotoxicity against tumor cells, secretion of antitumor cytokines, establishment of immunological memory, and modulation of other immune cells. However, whether this crucial effector population undergoes functional reprogramming under the influence of TGFβ1^+^ Treg during CRC progression remains an open question. To elucidate the dynamics of CD8^+^ T cells within the CRC immune landscape, we performed pseudotime trajectory analysis. The results demonstrated a progressive decline in CD8^+^ T cell abundance as differentiation advanced in the LDCA grade, particularly in the terminal grades, where the number of red dots markedly decreased (Figure 6A,B). This trend suggests that CD8^+^ T cells may undergo exhaustion or face constraints in persistence.

A deeper examination of gene expression patterns revealed characteristic functional deterioration in CD8^+^ T cells. Canonical exhaustion markers, such as TIGIT and LAG3, exhibited a progressive upregulation, whereas *CD8A* and *CD8B*, essential for cytolytic activity, displayed a marked decline. Additionally, immune regulatory genes, including *BATF* and *CD274*, were also downregulated (Figure 6C), indicating not only a loss of cytotoxic efficacy but also a diminished immunoregulatory capacity. To better visualize these alterations, we mapped the developmental trajectories of key gene expression patterns, depicting exhaustion induction, cytotoxic decline, and shifts in immunosuppressive function (Figure 6D). To ascertain whether this trend is consistently observed across different CRC grades, we compared the expression levels of CD8^+^ T cell signature genes in LDCA grade relative to HDCA and MDCA grades, juxtaposing these findings with the pseudotime trajectory results. The analysis revealed a significant upregulation of T cell exhaustion markers in LDCA, whereas genes associated with cytotoxicity and immune modulation were markedly downregulated (Figure S3). This convergence of findings reinforces the notion that during CRC progression, CD8^+^ T cells undergo a gradual shift from an active effector state to an exhausted phenotype. In order to delineate the functional transitions of CD8^+^ T cells across CRC grades, we conducted differential gene expression analysis and pathway enrichment on genes uniquely upregulated in LDCA grade (Figure 6E). LDCA‐grade CD8^+^ T cells were significantly enriched in pathways associated with immune suppression and cytotoxic impairment. Notably, the negative regulation of immune response pathway was prominently activated in LDCA‐grade CD8^+^ T cells, alongside the suppression of cytotoxicity‐associated pathways, including negative regulation of natural killer cell‐mediated cytotoxicity, negative regulation of natural killer cell‐mediated immunity, negative regulation of T cell‐mediated cytotoxicity, and negative regulation of leukocyte‐mediated cytotoxicity. Beyond the decline in cytotoxicity, CD8^+^ T cells in LDCA grade also exhibited heightened activation of apoptotic signaling. Several apoptosis‐related pathways, including the extrinsic apoptotic signaling pathway and the apoptotic signaling pathway, were significantly enriched (Figure 6F). These findings indicate that in advanced CRC, CD8^+^ T cells not only experience functional impairment but may also succumb to increased susceptibility to apoptosis, further exacerbating immune suppression and fostering conditions conducive to tumor immune evasion.

Collectively, these results depict a profound functional reprogramming of CD8^+^ T cells throughout CRC progression. As tumors advance to LDCA grade, CD8^+^ T cells progressively decline in number, exhibit diminished cytotoxicity, and shift toward an exhausted phenotype. The presence of TGFβ1^+^ Treg likely reinforces this transition by amplifying immunosuppressive signaling, accelerating CD8^+^ T cell exhaustion and functional attrition, thereby undermining antitumor immunity and facilitating CRC immune escape.

## 4 Discusssion

By integrating three distinct CRC scRNA‐seq datasets, this study provides deeper insights into the role of TGFβ1^+^ Tregs in CRC progression, extending beyond previous dataset analyses. In contrast to prior studies that primarily focused on overall immune cell populations or the composition of the immune microenvironment, our investigation emphasizes the dynamic changes and functional heterogeneity of TGFβ1^+^ Tregs across different tumor grades. The analysis suggests that TGFβ1^+^ Tregs may mediate CD8^+^ T cell dysfunction through specific ligand‐receptor interactions, potentially accelerating T cell exhaustion and apoptosis. This process is likely to contribute to the formation of a highly immunosuppressive tumor microenvironment, ultimately promoting CRC immune evasion and malignant progression. Through multi‐dataset integration and deep computational analysis, this study refines the understanding of TGFβ1^+^ Tregs in CRC immune suppression and identifies potential therapeutic targets, offering new directions for precision immunotherapy.

Analysis of the three datasets revealed that immune cell proportions exhibited more pronounced variations across tumor grades than tumor stages, suggesting that tumor differentiation status may play a more critical role in shaping the immune microenvironment (Figure 1). Interestingly, while many studies have focused on the impact of tumor stage on the microenvironment, with stage commonly used to predict patient prognosis and explain tumor behavior, our findings suggest that tumor grade also plays a crucial role in regulating the immune microenvironment in CRC. Although tumor stage, particularly in cases such as the pT3 stage, indeed alters the microenvironment through metabolic interactions between tumor cells and adipocytes, affecting immune cell infiltration and promoting tumor progression [49], we observed that the significant changes in immune cell proportions across different grades further highlight the potential importance of tumor grade in immune regulation. In the dynamic analysis, we found that the functional enrichment pathways of Treg cells in the late differentiation grade were primarily related to cell differentiation. Compared to the early differentiation grade of CRC, certain genes associated with poor CRC prognosis were significantly upregulated in the late grade, and Tregs actively responded to immunosuppressive and proliferative differentiation pathways during the later phases of tumor progression (Figure 2). Although the immunosuppressive role of Treg cells in the tumor microenvironment is well recognized, such as suppressing effector T cells and NK cell proliferation and activation through inhibitory cytokines (such as TGF‐β and IL‐10) [26, 50, 51], downregulating co‐stimulatory signals of antigen‐presenting cells via the CTLA‐4 pathway, consuming IL‐2 to limit effector T cell proliferation [41, 42, 43], and generating adenosine and inducing other immunosuppressive cells (such as, MDSCs) to form complex intercellular communication networks that promote tumor immune evasion and progression [52], little is known about the specific roles and changes of different Treg cell subsets.

Our study revealed significant changes in the proportions and functions of different Treg cell subsets within the tumor microenvironment during tumor progression. Specifically, in the late grade, the numbers of TGFβ1^+^ Tregs and HSP^+^ Tregs increased, with TGFβ1^+^ Tregs showing the most pronounced expansion, whereas the proportions of NCF1^+^ Tregs and proliferative Tregs decreased, reflecting a reduced demand for oxidative stress resistance and proliferation regulation (Figures 3 and 4). The pseudotime analysis further validated this trend, showing a significant increase in TGFβ1^+^ Tregs during the late tumor grade, while NCF1^+^ Tregs and proliferative Tregs decreased, a pattern opposite to that observed in the early pseudotime grades. Additionally, in the late grade, TGFβ1^+^ Tregs exhibited increased expression of immunosuppressive genes such as *PDCD1*, *CTLA4*, and *HAVCR2*, while proliferation‐related genes like *MKI67* and *PCNA* were also upregulated. These genes displayed consistent temporal expression changes within the corresponding Treg subsets, indicating a dynamic adjustment of immune regulatory mechanisms in the tumor microenvironment and underscoring the pivotal role of TGFβ1^+^ Tregs in immune evasion. This crucial role was further supported by our cell communication analysis, which revealed that TGFβ1^+^ Tregs engage in strong intercellular signaling through three key ligand‐receptor interaction, including KLRB1‐CLEC2D, LGALS9‐HAVCR2, and TNFSF10‐TNFRSF10B (Figure 5). KLRB1‐CLEC2D, LGALS9‐HAVCR2, and TNFSF10‐TNFRSF10B are three key receptor‐ligand pairs closely associated with cell apoptosis. The interaction between KLRB1 (encoding CD161) and its ligand CLEC2D modulates the activity of NK cells and T cells, potentially suppressing their cytotoxic functions under certain conditions, thereby facilitating tumor immune evasion [45]. In pan‐cancer research, this receptor‐ligand interaction has been recognized as an important regulatory axis [53]. However, studies on its specific role in individual cancer types remain scarce. The interaction between *LGALS9* (encoding Galectin‐9) and its receptor *HAVCR2* (TIM‐3) has been extensively demonstrated to suppress T cell function, induce T cell exhaustion, and reinforce an immunosuppressive microenvironment [54]. In colorectal cancer, upregulation of TIM‐3 and Galectin‐9 has been confirmed to correlate with tumor progression and poor prognosis [55]. Additionally, *TNFSF10* (encoding TRAIL) binds to its receptor *TNFRSF10B* (DR5) to induce apoptosis in tumor cells [56]. However, in many cancers, including colorectal cancer, TRAIL‐mediated apoptotic signaling can be inhibited, thereby promoting tumor survival [57]. As expected, CD8^+^ T cells in LDCA grade exhibit an upregulation of exhaustion‐related gene expression, a downregulation of cytotoxic function‐associated genes, and a decline in immune regulatory gene expression. Additionally, these cells actively respond to negative regulatory pathways of immune activation, while cytotoxic signaling pathways are also negatively modulated. This suggests that CD8^+^ T cells undergo progressive functional exhaustion during the later grades of CRC development. Tregs have a complex prognostic role in CRC, and research findings on this subject are somewhat controversial. However, growing evidence suggests that Tregs and their specific subsets play a crucial role in the initiation and progression of CRC. For instance, signaling mediated by Midkine (MDK) through Tregs promotes an immunosuppressive microenvironment and is associated with poor overall survival in CRC patients, particularly through the MDK/SDC4 axis [58]. Additionally, *Lactobacillus*‐derived ICA can inhibit the differentiation of CD4^+^ Tregs by modulating the IDO1/Kyn/AHR axis, thereby enhancing CD8^+^ T cell function and improving the efficacy of anti‐PD1 therapy in CRC, highlighting the potential of *Lactobacillus* as an adjuvant to enhance anti‐PD1 treatment [59]. The functional heterogeneity of Tregs is further reflected in specific subsets of helper T cells; for example, blocking interleukin‐17A in Tregs can restore the function of exhausted CD8^+^ T cells and inhibit CRC progression [59]. Moreover, our research has revealed that the TGFβ1^+^ Treg subset plays a pivotal role in tumor immune evasion and the exhaustion of CD8^+^ T cell function. These multidimensional pieces of evidence collectively highlight that, although the overall prognostic role of Tregs remains a subject of debate, a deeper understanding of their specific subsets and the molecular mechanisms they mediate is crucial for accurately assessing CRC prognosis and developing targeted therapeutic strategies.

Although our data reveal the role of TGFβ1^+^ Treg cells in tumor progression, we also recognize that the complexity of tumor progression can be better understood by comparing the characteristics of superficial tumors, the invasion front, and metastatic sites. The tumor microenvironment and its immune cell composition may differ significantly between these regions, and such differences may play a critical role in immune evasion and tumor progression. However, our study found significant differences in the composition of Treg cells in colorectal cancers with different grades, indicating that histological grading based on morphology conveys important biological information and provides unique insights into the immune regulatory mechanisms within the tumor microenvironment. Similarly, the immunosuppressive mechanisms mediated by Tregs have been extensively studied; most research has focused on their ability to suppress effector T cells and NK cells through the secretion of inhibitory cytokines. In contrast, this study highlights the potential role of Tregs in receptor‐ligand interactions within cell communication. However, scRNA‐seq‐based studies primarily rely on transcript‐level expression analysis and cannot directly assess the functional impact of these receptor‐ligand interactions at the protein level. Therefore, in future research, we will experimentally validate the role of key receptor‐ligand pairs to further confirm their function in Treg‐mediated immunosuppression and tumor microenvironment remodeling, providing a stronger foundation for precision‐targeted intervention strategies.

## 5 Conclusion

By analyzing three single‐cell datasets, we uncovered that TGFβ1^+^ Tregs orchestrate CD8^+^ T cell impairment through critical receptor‐ligand signaling, including LGALS9‐HAVCR2 and TNFSF10‐TNFRSF10B, thereby reinforcing immunosuppression in CRC. The involvement of TGFβ1^+^ Tregs in CRC immune evasion suggests their potential as a therapeutic target, providing new insights for the refinement of targeted immunotherapy strategies.

## Author Contributions

**Dongwei Li:** conceptualization (lead), methodology (lead), software (lead), writing – original draft (equal), writing – review and editing (supporting). **Jingya Liu:** data curation (equal), formal analysis (equal), investigation (equal), writing – review and editing (equal). **Lixian Yu:** supervision (equal), validation (lead), visualization (equal). **Xina Luo:** writing – review and editing (equal). **Xiaomei Wu:** supervision (lead), validation (equal), visualization (equal). **Ronghui Chen:** project administration (lead), resources (lead), writing – review and editing (equal).

## Consent

All authors approved the manuscript submitted for publication.

## Conflicts of Interest

The authors declare no conflicts of interest.

## Supporting information


**Data S1:** cam471202‐sup‐0001‐DataS1.docx.

## Data Availability

The data used in this study were obtained from the public database GEO, dataset numbers GSE164522, GSE132465, and GSE144735, which are publicly accessible to any researcher through the GEO database.
