# Supplementary material for: Integrated Single‐Cell RNA‐Seq Reveals Immunosuppressive Mechanisms of Treg Cell Differentiation and Tumor Microenvironment Interactions in Colorectal Cancer
Source: Cancer Med. 2025 Sep 2;14(17):e71202. doi: 10.1002/cam4.71202 (PMC12403112; doi:10.1002/cam4.71202)
Supplement: Supplementary file 1 — Data S1: cam471202‐sup‐0001‐DataS1.docx. [file CAM4-14-e71202-s001.docx]

Supplementary material: graphs and tables


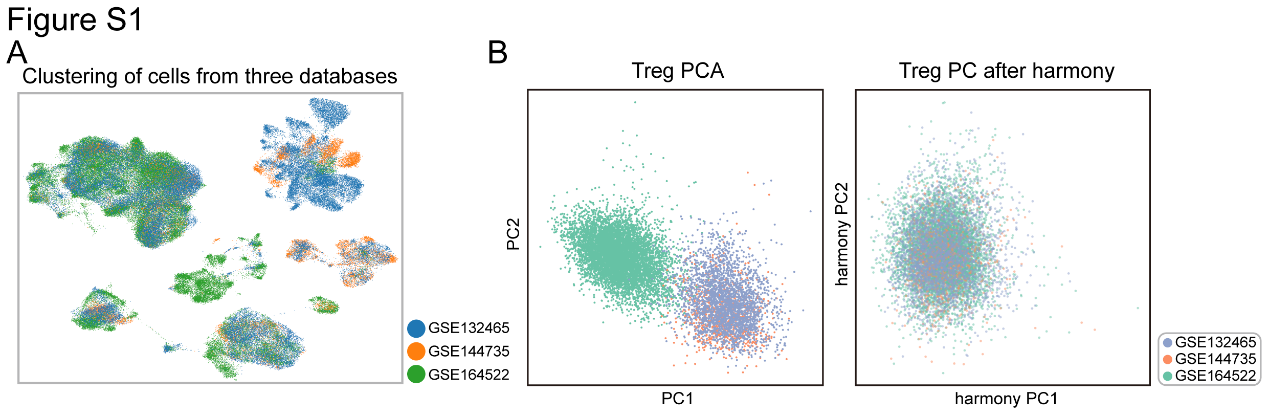


**FigS1. Integration and batch calibration of single cell RNA-seq datasets.**

A: Batch-calibrated UMAP plots of all cells from three independent datasets GSE132465 (blue), GSE144735 (orange), and GSE164522 (green). B: Treg batch-calibrated results from all datasets, with pre-calibration on the left, and post-calibration on the right.


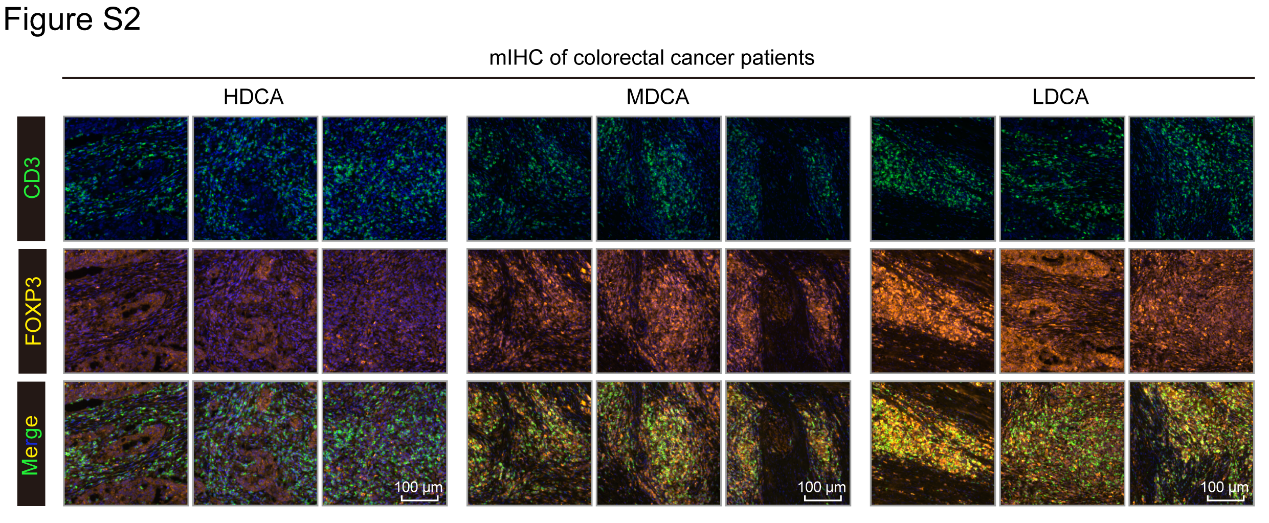
**Fig. S2. TGFβ1^+^ Treg increase in the LDCA grade.**

Representative immunofluorescence staining images of CRC patient tissue sections for each grade, where CD3 (green), FOXP3 (yellow), and DAPI (blue) are shown. Scale bar = 100 μm.


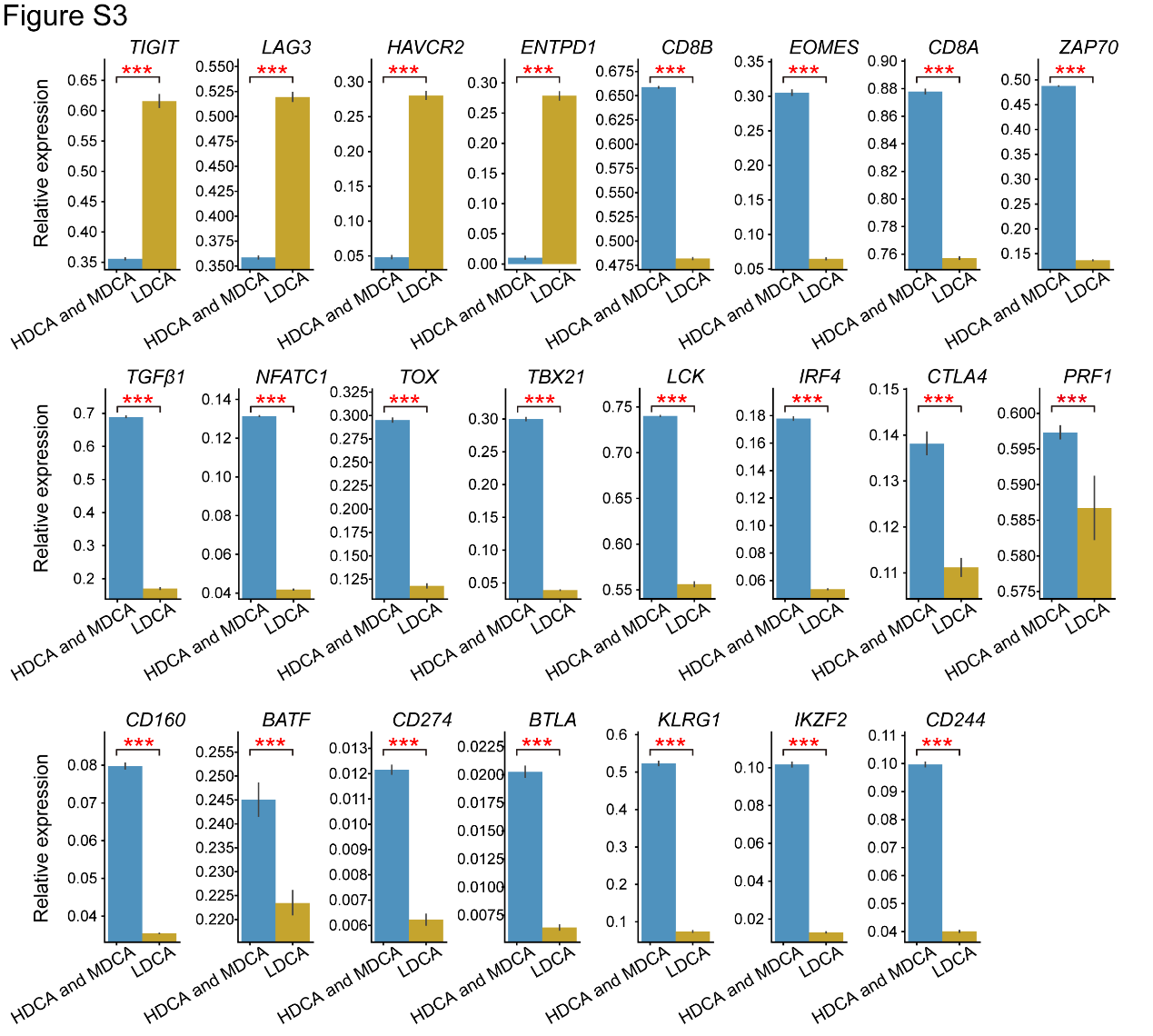
**Fig. S3. Relative expression of CD8^+^ T cell-related genes at different differentiation grades. ***, *p* < 0.001.**

**Table S1 Clinicopathological data of CRC patients from three GEO datasets**

| Patient No. | Gender | Age | Stage | Anatomic region | Grades |
| --- | --- | --- | --- | --- | --- |
| SMC01 | F | 64 | IIA | Adenocarcinoma | low-differentiated |
| SMC02 | M | 66 | IIIB | Adenocarcinoma | low-differentiated |
| SMC03 | F | 83 | IIIC | Adenocarcinoma | high differentiated |
| SMC04 | M | 69 | IIIB | Adenocarcinoma | moderate-differentiated |
| SMC05 | F | 58 | IIA | Adenocarcinoma | low-differentiated |
| SMC06 | M | 46 | IIIB | Adenocarcinoma | low-differentiated |
| SMC07 | F | 67 | I | Adenocarcinoma | low-differentiated |
| SMC08 | M | 68 | IIIB | Adenocarcinoma | moderate-differentiated |
| SMC09 | M | 75 | IIA | Adenocarcinoma | low-differentiated |
| SMC10 | F | 77 | IIA | Adenocarcinoma | moderate-differentiated |
| SMC11 | F | 38 | IIIA | Adenocarcinoma | low-differentiated |
| SMC14 | M | 77 | IIIB | Adenocarcinoma | moderate-differentiated |
| SMC15 | M | 56 | IIA | Adenocarcinoma | low-differentiated |
| SMC16 | M | 59 | IIIB | Adenocarcinoma | moderate-differentiated |
| SMC17 | M | 47 | IIIB | Mucinous Adenocarcinoma |  |
| SMC18 | F | 63 | IIA | Adenocarcinoma | moderate-differentiated |
| SMC19 | F | 80 | IIIB | Adenocarcinoma | moderate-differentiated |
| SMC20 | F | 65 | IIIB | Mucinous Adenocarcinoma |  |
| SMC21 | M | 51 | IVA | Adenocarcinoma | moderate-differentiated |
| SMC22 | M | 76 | IIIB | Adenocarcinoma | moderate-differentiated |
| SMC23 | F | 67 | IIIB | Adenocarcinoma | moderate-differentiated |
| SMC24 | F | 48 | I | Adenocarcinoma | low-differentiated |
| SMC25 | F | 57 | IVA | Adenocarcinoma | moderate-differentiated |
| KUL01 | F | 81 | IIB | Adenocarcinoma | moderate-differentiated |
| KUL19 | F | 86 | IIIB | Adenocarcinoma | moderate-differentiated |
| KUL21 | F | 50 | IVA | Adenocarcinoma | moderate-differentiated |
| KUL28 | M | 52 | IIA | Adenocarcinoma | moderate-differentiated |
| KUL30 | M | 84 | IIA | Adenocarcinoma | moderate-differentiated |
| KUL31 | M | 85 | I | Adenocarcinoma | low-differentiated |
| CRLM01 | M | 62 | IVA | Neuroendocrine | G2 |
| CRLM02 | F | 67 | IVA | Adenocarcinoma | low or moderate-differentiated |
| CRLM03 | M | 63 | IVA | Adenocarcinoma | moderate-differentiated |
| CRLM04 | M | 47 | IVA | Adenocarcinoma | high or moderate-differentiated |
| CRLM05 | M | 61 | IVA | Neuroendocrine | G2 |
| CRLM06 | F | 47 | IVA | Adenocarcinoma | moderate-differentiated |
| CRLM07 | F | 52 | IVA | Adenocarcinoma | low-differentiated |
| CRLM08 | M | 68 | IIIB | Adenocarcinoma | moderate-differentiated |
| CRLM09 | M | 63 | IVA | Adenocarcinoma | moderate-differentiated |
| CRLM10 | M | 75 | IVA | Adenocarcinoma | moderate-differentiated |
| CRLM11 | F | 60 | IVA | Adenocarcinoma | moderate-differentiated |
| CRLM12 | M | 53 | IVA | Adenocarcinoma | moderate-differentiated |
| CRLM13 | F | 79 | IVA | Adenocarcinoma | moderate-differentiated |
| CRLM14 | M | 53 | IVA | Adenocarcinoma | moderate-differentiated |
| CRLM15 | F | 67 | IVA | Adenocarcinoma | low-differentiated |
| CRLM16 | M | 45 | IVA | Adenocarcinoma | low-differentiated |
| CRLM17 | M | 73 | IVA | Adenocarcinoma | low or moderate-differentiated |

Note: We ultimately excluded the data of patients with a small sample size and no staging information, specifically SMC17, SMC20, CRLM01, and CRLM05. Data from 43 other patients were included in the analysis.
